# Ultralow Lattice Thermal Conductivity and Improved Thermoelectric Performance in Cl-Doped Bi_2_Te_3–*x*_Se_*x*_ Alloys

**DOI:** 10.1021/acsami.2c08686

**Published:** 2022-07-13

**Authors:** Taras Parashchuk, Rafal Knura, Oleksandr Cherniushok, Krzysztof T. Wojciechowski

**Affiliations:** † Thermoelectric Research Laboratory, Department of Inorganic Chemistry, Faculty of Materials Science and Ceramics, AGH University of Science and Technology, Mickiewicza Ave. 30, Krakow 30-059, Poland; ‡ Department of Science, Graduate School of Science and Technology, Kumamoto University, 2 Chome-39-1 Kurokami, Chuo Ward, Kumamoto 860-8555, Japan

**Keywords:** bismuth telluride, thermoelectric properties, anisotropy, bipolar conduction, two-band Kane model, bonding inhomogeneity, lone-pair electrons

## Abstract

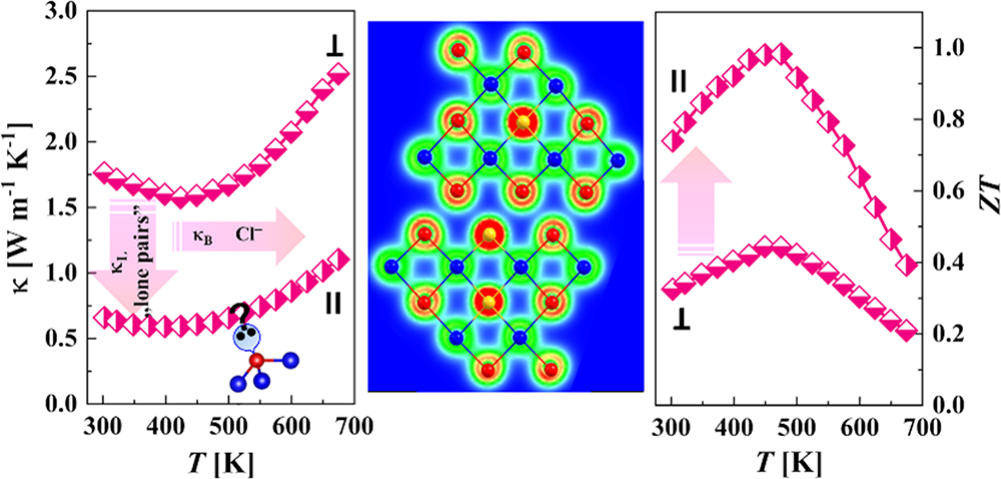

Bi_2_Te_3_-based alloys are the main materials for the construction of low- and medium-temperature thermoelectric modules. In this work, the microstructure and thermoelectric properties of Cl-doped Bi_2_Te_3–*x*_Se_*x*_ alloys were systematically investigated considering the high anisotropy inherent in these materials. The prepared samples have a highly oriented microstructure morphology, which results in very different thermal transport properties in two pressing directions. To accurately separate the lattice, electronic, and bipolar components of the thermal conductivity over the entire temperature range, we employed a two-band Kane model to the Cl-doped Bi_2_Te_3–*x*_Se_*x*_ alloys. It was established that Cl atoms act as electron donors, which tune the carrier concentration and effectively suppress the minority carrier transport in Bi_2_Te_3–*x*_Se_*x*_ alloys. The estimated value of the lattice thermal conductivity was found to be as low as 0.15 Wm^–1^ K^–1^ for Bi_2_Te_3–*x*–*y*_Se_*x*_Cl_*y*_ with *x* = 0.6 and *y* = 0.015 at 673 K in parallel to the pressing direction, which is among the lowest values reported for crystalline materials. The large reduction of the lattice thermal conductivity in both pressing directions for the investigated Bi_2_Te_3–*x*_Se_*x*_ alloys is connected with the different polarities of the Bi-(Te/Se)1 and Bi-(Te/Se)2 bonds, while the lone-pair (Te/Se) interactions are mainly responsible for the extremely low lattice thermal conductivity in the parallel direction. As a result of the enhanced power factor, suppressed bipolar conduction, and ultralow lattice thermal conductivity, a maximum ZT of 1.0 at 473 K has been received in the Bi_2_Te_2.385_Se_0.6_Cl_0.015_ sample.

## 1 Introduction

Understanding and control of the thermal transport in thermoelectric (TE) materials can significantly improve the ability to interconvert heat and electricity by TE devices.^1,2^ The performance of the TE materials is represented by a dimensionless figure of merit ZT = σ*S*^2^*T*/(κ_L_ + κ_e_ + κ_B_), where σ is the electrical conductivity; *S* is the Seebeck coefficient; κ_L_, κ_e_, and κ_B_ are the lattice, electronic, and bipolar components of the thermal conductivity, respectively; and *T* is the absolute temperature. Due to the interlink between electronic thermal conductivity κ_e_ with the electrical conductivity σ through the Wiedemann–Franz law (κ_e_ = *L*σ*T*, where *L* is the Lorenz number), a good way to improve the TE performance of materials is connected with the decrease of κ_L_ and κ_B_.

The lattice component of the thermal conductivity can be suppressed by the fabrication of solid solutions,^3^ grain boundaries engineering,^4^ micro- and nano-inclusions,^5^ and point defects.^6^ However, the mentioned approaches usually reflect another important parameter, carrier mobility μ, which causes the low power factor (PF = *S*^2^σ) and deteriorates the thermoelectric performance of materials.^7^ Therefore, finding a way to disturb the phonon transport without a negative effect on electronic transport is among the main tasks in modern thermoelectric science.

Recently discovered novel approaches to reduce κ_L_ are mainly connected with the complexities of the crystal structure. It was shown that such effects as lattice anharmonicity, reflected in high Grüneisen parameters;^8,9^ lone pair electrons;^10,11^ resonant bonding;^12,13^ rattling atoms in a frame of ″phonon-glass and electron-crystal″ (PGEC) concept;^14^ liquid-like behavior of superionic conductors described by ″phonon-liquid and electron-crystal″ (PLEC) concept;^15−17^ and the newly developed theory of bonding inhomogeneity^18−20^ are the main crystal structure indicators of low lattice thermal conductivity.

For optimal power factor over a broad temperature range, the TE materials should have an attuned band gap, usually in the range of 6–10*k*_B_*T* (*k*_B_ is the Boltzman constant).^21,22^ For such narrow-band-gap materials, the transport of minority carriers at elevated temperatures is one of the main obstacles, which dramatically decreases the ZT parameter due to the lowering of the Seebeck coefficient and enhancement of the bipolar thermal conductivity. Therefore, the suppression of the intrinsic transport is greatly in line with the improvement of the ZT parameter over a broad temperature range and shall significantly facilitate the energy conversion efficiency.

Bismuth telluride-based alloys are the most commercialized thermoelectric materials, which are intensively used for the construction of the TE modules.^23,24^ A set of unique properties, i.e., narrow band gap, high dielectric constant, multivalley band structure, the high solubility of dopants, and excellent mechanical properties, makes this material irreplaceable for TE applications.^25−31^ Single-crystalline Bi_2_Te_3_-based alloys exhibit high anisotropy of the transport properties due to a layered crystal structure. This material consists of the quintuple layers with Bi–Te covalent bonds separated by the weak van der Waals interactions.^32−34^ Properties of the materials are strongly dependent on the direction of the crystallization. Polycrystalline Bi_2_Te_3_-based materials usually are also highly oriented.^33,35^ Depending on the direction of the measurement (along or across the quintuple layers), Hellman and Broido predicted a large difference in the lattice thermal conductivity for stoichiometric Bi_2_Te_3._^36^ Grin explains this as the presence of the large bonding inhomogeneity and lone-pair interaction, which reduce the lattice thermal conductivity.^18^ An even larger effect on the phonon transport in different crystallization directions is expected in *p*-Bi_2-*x*_Sb_*x*_Te_3_ and *n*-Bi_2_Te_3–*x*_Se_*x*_ alloys, which have a special practical interest in module construction. It should be mentioned that the reported ZT parameter for *p*-type Bi_2–*x*_Sb_*x*_Te_3_^37^ significantly exceeds the TE performance of the *n*-type Bi_2_Te_3–*x*_Se_*x*_ counterpart.^38,39^ Therefore, the optimization of the electrical and thermal transport of these *n*-type alloys is highly challenging and desired.

Keeping in mind that the TE performance of Bi_2_Te_3_-based alloys is strongly affected by the lattice κ_L_ and bipolar κ_B_ components of the thermal conductivity, in this work, we engineer both parameters simultaneously. With this aim, we systematically studied the microstructure and TE properties of the Cl-doped Bi_2_Te_3–*x*_Se_*x*_ alloys. As anisotropy is known to be an important factor that affects the transport properties of the Bi_2_Te_3_-based alloys, our special attention was also dedicated to this problem. The investigated samples were found to be strongly anisotropic considering the pressing direction; therefore, all physical properties were studied in parallel and perpendicularly to the pressing direction. Chlorine was selected as a dopant due to its expected donor effect on the electronic properties and possible suppression of the bipolar thermal conductivity. To accurately determine the Cl effect on the transport properties of the investigated *n*-type Bi_2_Te_3–*x*_Se_*x*_ alloys, we adopted the two-band Kane model. As a result of the optimized power factor and reduced thermal conductivity, the TE figure of merit ZT reaches a maximum of 1.0 at 473 K for *n*-type Bi_2_Te_3–*x*–*y*_Se_*x*_Cl_*y*_, (*x* = 0.6, *y* = 0.015). Moreover, due to the attuned chemical potential and effective band gap engineering, the ZT shows the maximum value at different operating temperatures, opening the potential of these materials for the construction of the functionally graded TE legs. The main reasons for the excellent TE performance of the investigated alloys are connected with the extremely low lattice thermal conductivity κ_L_ (as low as 0.15 Wm^–1^ K^–1^ at 673 K) and effective reduction of the bipolar thermal conductivity κ_B_. The intrinsic transport was effectively suppressed due to the attuned band gap through Bi_2_Te_3_-Bi_2_Se_3_ alloying and chemical potential engineering induced by the Cl dopant. The DFT calculations proved that the reduction of κ_L_ is connected with the large bonding inhomogeneity of the Bi-(Te/Se)1 and Bi-(Te/Se)2 bonds, as well as the lone-pair interactions of the Te/Se atoms.

## 2 Methods

### 2.1 Synthesis and Characterization of Materials

The synthesis of materials was carried out in graphite-coated quartz ampoules evacuated to a residual pressure of 10^–5^ mbar. Polycrystalline Bi_2_Te_3–*x*–*y*_Se_*x*_Cl_*y*_ (*x* = 0.6, *y* = 0.015; *x* = 0.6, *y* = 0.03; *x* = 0.3, *y* = 0.015; *x* = 0.3, *y* = 0.03; and *x* = 0.6, *y* = 0) specimens were synthesized by melting the elements Bi (Alfa Aesar, 99.999%), Te (Alfa Aesar, 99.999%), Se (Alfa Aesar, 99.999%), and BiCl_3_ (Alfa Aesar, 99.999%)) at 1123 K in a rocking furnace and then quenched in cold water. The resultant ingots were crushed into fine powders by ball milling and densified by the spark plasma sintering (SPS) technique at 673 K for 10 min in a 12.7 mm diameter graphite mold under an axial compressive stress of 45 MPa in an argon atmosphere. The heating/cooling rate was 50 K/min.

**Figure 1:**
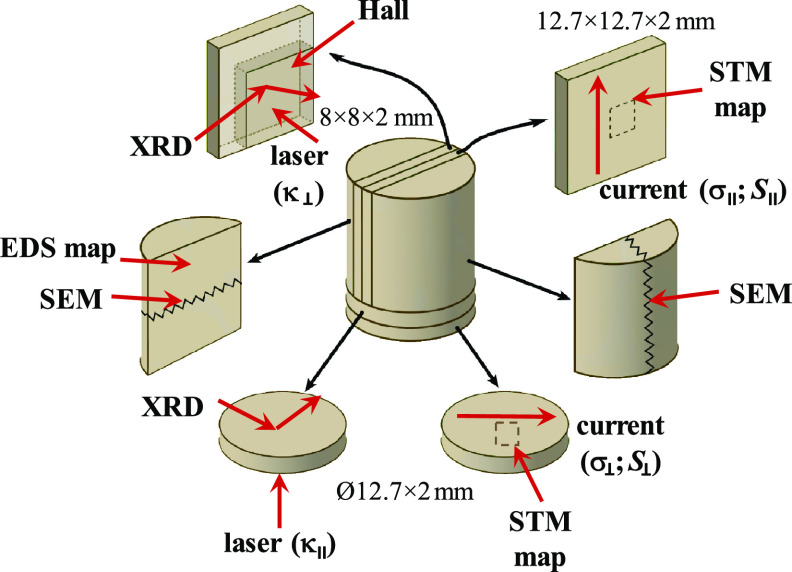
Preparation of samples for the characterization of physical properties in different pressing directions.

Highly dense (>98% of crystallographic density) cylinders with a diameter of 12.7 mm and length of ∼17 mm were obtained and cut for further characterization. To investigate the physical properties in parallel and perpendicular to the pressing direction, two series of samples were cut from each cylinder for the transport properties measurements, as depicted in Figure 1.

Phase identification was performed with a Bruker D8 Advance X-ray diffractometer using Cu Kα radiation (λ = 1.5418 Å, Δ2θ = 0.007°, 2θ range 10–100°) with Bragg–Brentano geometry. The positions of reflections, obtained by profile deconvolution, were corrected using the internal standard LaB_6_ (*a* = 4.15692(1) Å). The lattice parameters were accurately determined by the least-squares refinement using the WinCSD program package.^40^

For SEM and EDS analyses, samples were embedded in a conductive resin and polished using 0.1 μm diamond powder in a slurry. The analysis of the sample chemical composition was performed using scanning electron microscopy (JEOL JSM-6460LV Scanning Electron Microscope) equipped with energy-dispersive X-ray spectroscopy. The distribution of the Seebeck coefficient on the sample’s surface was analyzed using the scanning thermoelectric microprobe (STMp) technique with a resolution of 50 μm as well as a scanning thermoelectric microscope (STM) with a resolution of 1 μm. The measurements were carried out at 298 K.

### 2.2 Measurements of Electrical and Thermal Transport Properties

The Hall effect was investigated by the four-probe method in constant electrical and magnetic fields with the magnetic field induction of 0.9 T and current of 500 mA using homemade equipment. The estimated uncertainty of the Hall measurements was ∼10%.

The Seebeck coefficient *S* and electrical conductivity σ were measured by the commercial apparatus Netzsch SBA 458 Nemesis. Measurements were performed in an argon flow over the temperature range of 298–673 K. Thermal diffusivity α was measured by the Netzsch LFA 457 equipment, and the specific heat capacity *C*_p_ was estimated using the Dulong–Petit limit. The samples were first spray-coated with a thin layer of graphite to minimize errors from the emissivity of the material and laser beam reflection caused by a shiny pellet surface. Thermal conductivity was calculated using the equation κ *=* ρ*C*_p_α, where ρ is the density obtained by the Archimedes principle at the specimens from SPS. The speed of sound was measured at room temperature using the ultrasonic flaw detector Olympus Epoch 650. The uncertainty of the Seebeck coefficient and electrical conductivity measurements was 6%; the uncertainty of the thermal diffusivity measurements was 3%. The combined uncertainty for the determination of the dimensionless thermoelectric figure of merit ZT was ∼20%.

### 2.3 Computational Details

Quantum chemical (QC) calculations were performed using the Firefly QC program package,^41^ which is based on the GAMESS (US) source code.^42^ The calculations were performed based on the hybrid functional B3LYP that uses the Becke GGA functional for the exchange energy and the Lee–Yang–Parr GGA functional for the correlation energy. For the calculations, we used lattice parameters, symmetry information, and atomic coordinates available in the literature for the Bi_2_Te_3_ compound (ICSD #74348).^43^ The basis sets for the self-consistent calculations can be obtained from the authors. The analysis of the chemical bonding for the investigated materials was performed by the electron localizability approach. For this purpose, the electron density maps were calculated and visualized with the specialized module implemented in the ChemCraft software.^44^

## 3 Results and Discussion

### 3.1 Phase and Microstructural Analysis

**Figure 2:**
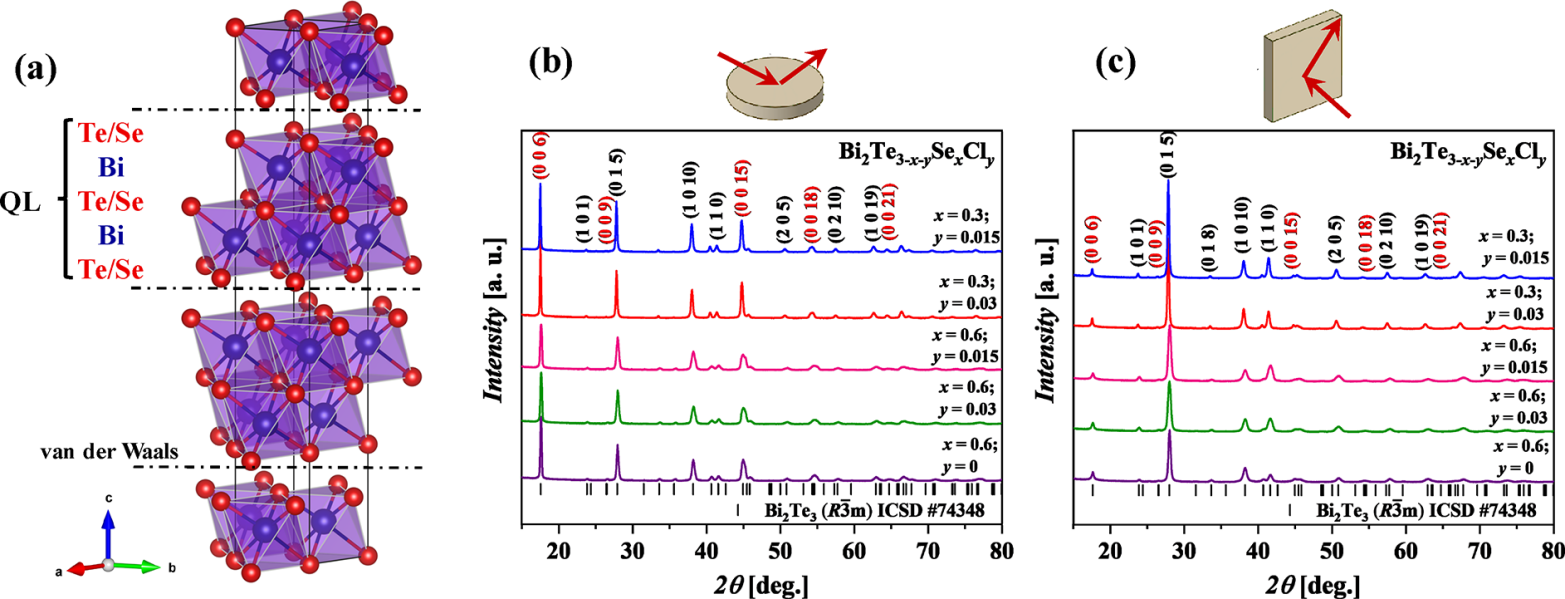
(a) Crystal structure of the Bi_2_Te_3_/Bi_2_Se_3_ solid solution characterized by Te/Se-Bi-Te/Se-Bi-Te/Se quintuple layers and van der Waals gaps in between. (b, c) X-ray diffraction patterns of the SPS-prepared Bi_2_Te_3–*x*–*y*_Se_*x*_Cl_*y*_ specimens measured in different pressing directions as shown in the figures.

To analyze the texturization of the sample morphology, the X-ray diffraction (XRD) patterns were recorded in parallel and perpendicularly to the pressure direction for all investigated polycrystalline samples (Figure 2). The rhombohedral structure of Bi_2_Te_3_ (ICSD #74348) was used to index all observed reflections. The relatively large breadth of the observed diffraction peaks is typical for solid solutions. No impurity phases were detected.

Powder XRD patterns collected on the surfaces perpendicular to the pressure direction show that the relative intensities of the basal planes (00*l*), in particular the (006), (0015), (0018), and (0021) planes, are much higher compared to the standard pattern of Bi_2_Te_3_ (ICSD #74348) (Figure 2b). To quantify the degree of preferred orientation *F* of the (00*l*) planes, the Lotgering method^45^ was applied using the following equations:
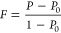
1
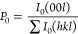
2
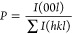
3where *P* is the ratio of the integral intensities of the (00*l*) planes to the intensities of the (*hkl*) planes in anisotropic samples and *P*_0_ is the ratio of the integral intensities of the (00*l*) planes to the intensities of the (*hkl*) planes in isotropic material. *I* and *I*_0_ are the intensities of the diffraction reflections of the measured samples and the standard isotropic Bi_2_Te_3_ (ICSD #74348), respectively. The (006), (0015), (0018), (0021), and (0024) reflections were chosen as *I*(00*l*) and *I*_0_(00*l*); at the same time, all the visible reflections in the XRD patterns were used for *I*(*hkl*) and *I*_0_(*hkl*) calculation. Integral intensities of all detected reflections were determined from powder pattern deconvolution in the WinCSD program package. Obtained results are shown in Table 1; the estimated values of *F* are within the range of 0.28–0.36, which are comparable to the results obtained using hot-pressing^46^ or hot-extrusion^47^ (*F* = 0.1–0.37). The higher degree of preferred orientation for the Cl-free sample (*F* = 0.36) in comparison to Cl-doped samples (*F* = 0.28–0.30) may be connected with the violated growth of grains in certain directions due to Cl-injected substitutional point defects. Moreover, the complex behavior of halogen atoms in Bi_2_Te_3_ reflected in the possible appearance of interstitial defects in van der Waals gaps may also decrease the degree of preferred orientation.^33^

**Table 1:** Degree of Preferred Orientation *F* of the (00*l*) Planes for Bi_2_Te_3–*x*–*y*_Se_*x*_Cl_*y*_ Specimens Measured Perpendicularly to the Pressing Direction and Lattice Parameters of Powdered Ingots after Synthesis Determined with the LaB_6_ Standard

Bi_2_Te_3–*x*–*y*_Se_*x*_Cl_*y*_	*F*	*a*, Å	*c,* Å
*x* = 0.6; *y* = 0	0.36	4.354(9)	30.16(7)
*x* = 0.3; *y* = 0.03	0.30	4.354(1)	30.297(7)
*x* = 0.3; *y* = 0.015	0.28	4.3593(8)	30.369(6)
*x* = 0.6; *y* = 0.03	0.30	4.341(7)	29.98(8)
*x* = 0.6; *y* = 0.015	0.29	4.342(6)	30.14(6)

The lattice parameters of Bi_2_Te_3–*x*–*y*_Se_*x*_Cl_*y*_ powdered ingots (Figure S1) after synthesis were accurately determined by the least-squares refinement using the WinCSD program package, and the results are listed in Table 1. Compared to the pure Bi_2_Te_3_ (*a* = 4.395(3) Å, *c* = 30.440(10) Å; ICSD #74348), all Se substituted samples have smaller lattice parameters due to the smaller ionic radius of Se^2–^ (1.98 Å) in comparison to Te^2–^ (2.21 Å).^48^ The Cl-doped samples show even lower lattice parameters, which are connected with a smaller ionic radius of this element (1.81 Å). This observation can be an indicator of the successful substitution of (Te/Se) by Cl atoms.

**Figure 3:**
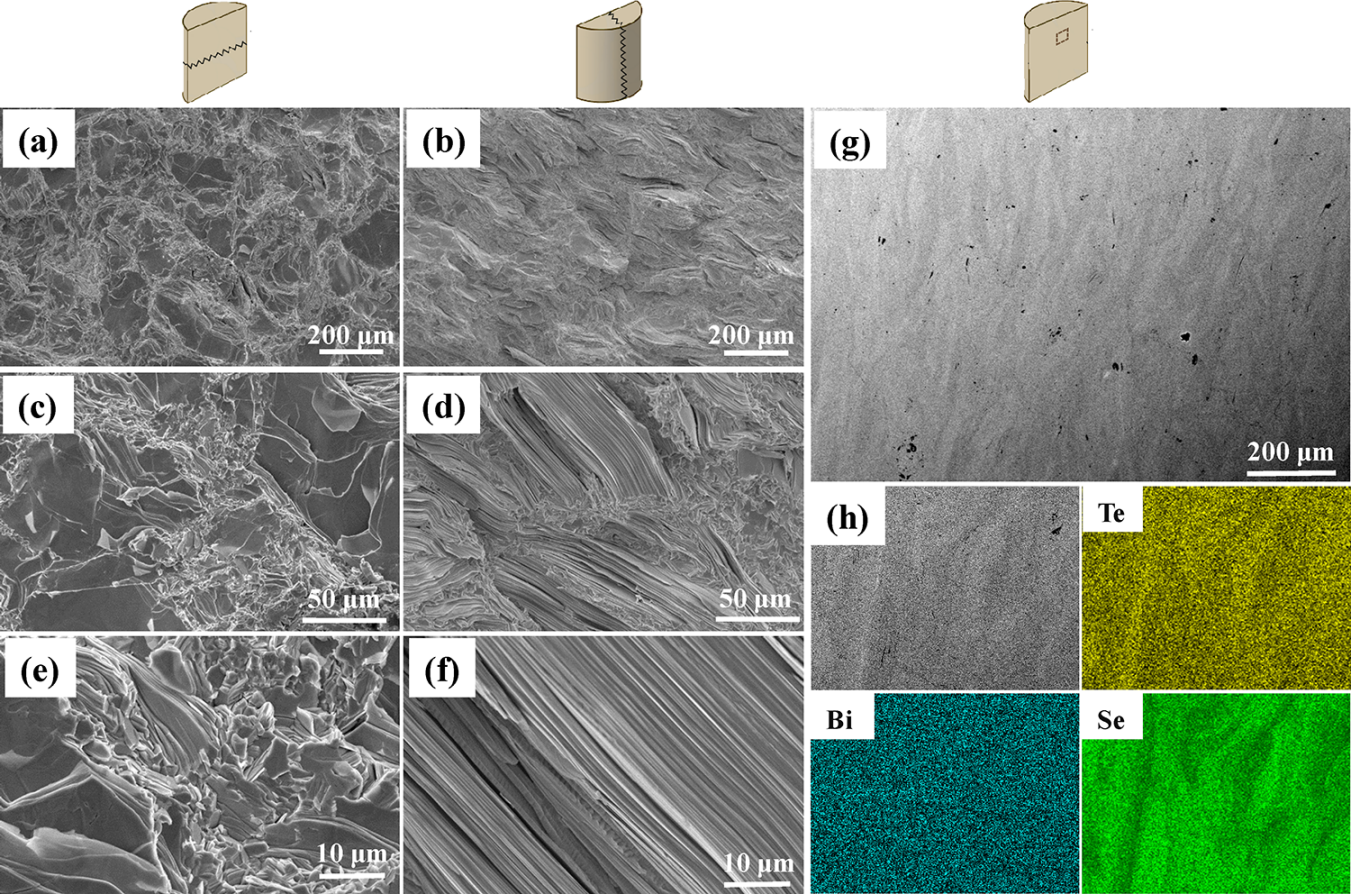
(a–f) Secondary electron images of the fractured surface of the SPS-prepared Bi_2_Te_2.385_Se_0.6_Cl_0.015_ specimen scanned in different pressing directions as shown in the figures. (g) Backscattered electron image for the polished surface of the SPS-prepared Bi_2_Te_2.385_Se_0.6_Cl_0.015_ specimen. (h) EDS elemental mapping for the SPS-prepared Bi_2_Te_2.385_Se_0.6_Cl_0.015_ specimen.

**Figure 4:**
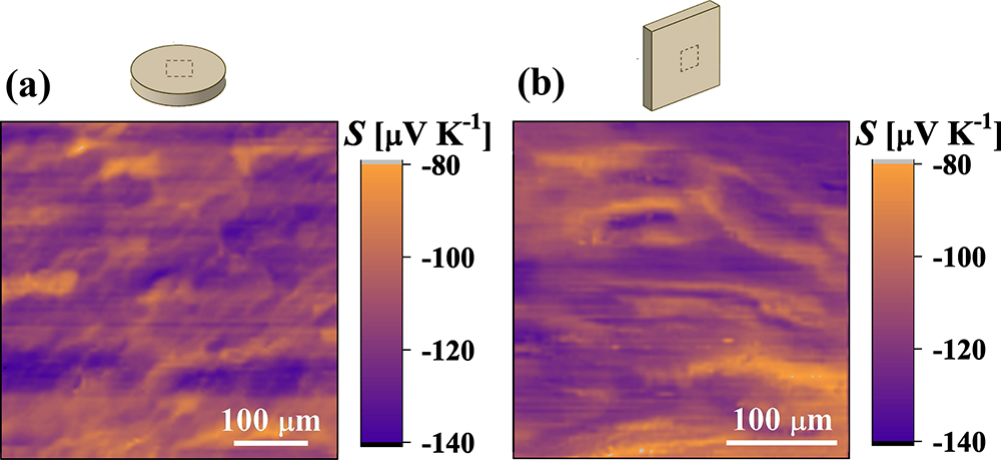
Spatial distribution of the Seebeck coefficient on the polished surface of the SPS-prepared Bi_2_Te_2.685_Se_0.3_Cl_0.015_ specimen scanned (a) in parallel and (b) perpendicularly to the pressing directions as shown in the figures.

In line with the XRD data, the provided SEM analysis confirms that the materials do not contain any impurities. Although the EDS mapping indicates that Bi is homogeneously distributed within the sample surface, a slightly inhomogeneous distribution of Te and Se was detected (Figure 3g,h). However, such inhomogeneities are common for solid solutions, and long-term homogenization annealing probably would eliminate this effect. In agreement with the XRD data, obvious grain orientations are also observed on the fractured surfaces for the Bi_2_Te_2.385_Se_0.6_Cl_0.015_ sample, as shown in Figure 3a–f. These results demonstrate that strong texturization is formed in the bulk samples after the SPS procedure and suggest a large difference in transport properties measured in different directions. The distribution of the Seebeck coefficient on the sample’s surface obtained using the scanning thermoelectric microscope with a spatial resolution of 1 μm also suggests the different orientations of grains in different pressing directions, as depicted in Figure 4a,b.

The spatial distribution of the Seebeck coefficient *S* for Bi_2_Te_3–*x*–*y*_Se_*x*_Cl_*y*_ (*x* = 0.6, *y* = 0.015; *x* = 0.6, *y* = 0.03; *x* = 0.3, *y* = 0.015; *x* = 0.3, *y* = 0.03; and *x* = 0.6, *y* = 0) specimens measured using the scanning thermoelectric microprobe is depicted in Figure S2a–e. The histograms of the Seebeck coefficient *S* across the sample surface have been fitted using a Gaussian distribution function. A standard deviation (SD) value was chosen as a parameter that represents spatial uniformity. The SD value of the Seebeck coefficient distribution for the investigated samples is in the range of 8–14 μV/K, indicating the high spatial homogeneity of the Bi_2_Te_3–*x*–*y*_Se_*x*_Cl_*y*_ specimens. The highest standard deviation (the lowest spatial homogeneity) of ∼14 μV/K was obtained for the sample that was not doped with chlorine, while Cl-doped specimens show the highest spatial homogeneity. The main reason for the high spatial homogeneity for heavily Cl-doped samples is connected with the higher carrier concentration (Table 2) of these samples and the deep location of the chemical potential of electrons in the conduction band. In this case, the chemical inhomogeneities, which are inherent in the polycrystalline samples, do not cause a large difference in the Seebeck coefficient. On the other hand, in the lighter Cl-doped or undoped samples, the chemical potential is located closer to the band gap. As a result, the minor difference in the chemical content of samples results in the movement of the chemical potential near the band gap and a large deviation of the Seebeck coefficient. For narrow band gap semiconductors, this effect can even lead to both *n*- and *p*-type conduction on the surface of one sample.^17,49^ The average Seebeck coefficient values are lower (65–70 μV/K) for the heavier Cl-doped samples and higher (118–155 μV/K) for the lighter Cl-doped specimens. The scanning thermoelectric microprobe analysis highlights the positive effect of Cl on the spatial homogeneity of the prepared samples.

**Table 2:** Seebeck Coefficient *S*, Electrical Conductivity σ, Thermal Conductivity κ, Hall Charge Carrier Concentration *n*_H_, Carrier Mobility μ*,* and DOS Effective Mass m* for Bi_2_Te_3–*x*–*y*_Se_*x*_Cl_*y*_ Specimens at Room Temperature

Bi_2_Te_3–*x*–*y*_Se_*x*_Cl_*y*_	*S*, μV K^–1^	σ, S cm^–1^	κ, Wm^–1^ K^–1^	*n*_,_ cm^–3^	μ, cm^2^ V^–1^ s^–1^	m*/*m*_e_
*x* = 0.6; *y* = 0	–144	390	0.70	5.8 × 10^19^	42	1.24
*x* = 0.3; *y* = 0.03	–78	2200	1.39	1.5 × 10^20^	92	0.91
*x* = 0.3; *y* = 0.015	–117	1160	1.00	7.5 × 10^19^	97	1.09
*x* = 0.6; *y* = 0.03	–61	1930	1.42	1.8 × 10^20^	67	0.64
*x* = 0.6; *y* = 0.015	–148	740	0.66	7.0 × 10^19^	66	1.48

### 3.2 Electrical and Thermal Transport Properties

In this work, two series of Bi_2_Te_3–*x*_Se_*x*_ samples with *x* = 0.3 and *x* = 0.6 (corresponding to the 90 mol % Bi_2_Te_3_–10 mol % Bi_2_Se_3_ and 80 mol % Bi_2_Te_3_–20 mol % Bi_2_Se_3_ compositions of the solid solution) were investigated. Such chemical composition of samples suggests a low lattice thermal conductivity (Figure S3a, Supporting information), which is one of the requirements for highly efficient thermoelectric materials.^21^ On the other hand, the alloying of Bi_2_Te_3_ with Bi_2_Se_3_ allows the opening band gap *E*_g_ from 0.13 eV for undoped Bi_2_Te_3_ to the values of 0.2 and 0.25 eV for Bi_2_Te_3–*x*_Se_*x*_ with *x* = 0.3 and *x* = 0.6, respectively (Figure S3b, Supporting information).^33^ This should deteriorate the minority carrier transport, which is typical for undoped Bi_2_Te_3_ even at room temperature. The compromise between the low lattice thermal conductivity and large band gap was the main criterion for the selection of chemical composition in the investigated Bi_2_Te_3–*x*_Se_*x*_.

Chlorine as a doping element in Bi_2_Te_3–*x*_Se_*x*_ alloys was chosen due to the following reasons. Halogen atoms (iodine, chlorine, and bromine) in the investigated alloys are known to be donor impurities.^50−52^ The number of electrons in the valence shell of halogen atoms is one more than that in tellurium atoms; therefore, a halogen atom can donate one electron to the conduction band. Moreover, the interaction of this electron with the ionized halogen atom will be weakened due to the strong influence of the polarization of media as a result of the high dielectric constant (ε = 80);^33^ hence, the negative effect of halogen doping on the carrier mobility μ should not be significant. Among other halogens, Birkhoz and Haacke reported that the Cl dopant shows the lowest effect on the carrier mobility μ in Bi_2_Te_3_ crystals,^53^ making this dopant promising for attuning transport properties in the Bi_2_Te_3–*x*_Se_*x*_ alloys.

To verify the assumption about the effect of Cl on the transport properties of Bi_2_Te_3–*x*_Se_*x*_ alloys, the Hall measurements were carried out and compared with the TE properties. The Seebeck coefficient *S*, electrical conductivity σ, and thermal conductivity κ, as well as the measured Hall mobility μ and Hall concentration *n* at room temperature, are shown in Table 2. The values of the Seebeck coefficient correspond well with the average *S* values recorded using the scanning thermoelectric microprobe technique. The increase of the nominal concentration of chlorine in Bi_2_Te_3–*x*–*y*_Se_*x*_Cl_*y*_ samples from *y* = 0.015 to 0.03 increases the value of the carrier concentration from 7.0–7.5 × 10^19^ to 1.5–1.8 × 10^20^ cm^–3^, leading to an approximately 2-fold drop in the absolute value of the Seebeck coefficient. The values of electrical conductivity are also significantly higher for the specimens with a larger content of Cl due to the increase in carrier concentrations.

The carrier mobility of the Cl-doped samples shows values in the range of 66–92 cm^2^ V^–1^ s^–1^, which are higher compared with the value of 41 cm^2^ V^–1^ s^–1^ recorded for the Cl-free sample. The enhancement of carrier mobility μ comes together with the increase in carrier concentration *n*. Particularly, Cl-doped samples show higher carrier mobility compared with the undoped sample (Table 2). A similar phenomenon was also observed for I-doped Bi_2_Te_3–*x*_Se_*x*_ materials by Kim et al.^50^ and Hong et al.^54^ While Hong et al. did not highlight the reason for the enhanced mobility by halogen doping, Kim et al. connected this effect with the larger mean free path of the carriers due to reduction point defects in halogen-doped Bi_2_Te_3–*x*_Se_*x*_ materials. Let us discuss this effect in more detail for the case of Cl-doped Bi_2_Te_3–*x*_Se_*x*_. Bi_2_Te_3_ and Bi_2_Se_3_ compounds crystallize with the deviation of stochiometry with an excess of metal.^33^ The evaporation of consisting elements is the main reason for the formation of vacancies, and the motivation of the antisite defects is the differences in electronegativity and size of atoms.^54^ While Se vacancies are reported to be the dominant defects in Bi_2_Se_3_, the antisite defects of Bi in Te sites and Te vacancies are the dominant defects in Bi_2_Te_3._^54,55^ The formation of the Bi_2_Te_3_-Bi_2_Se_3_ solid solution probably leads to the combination of both types of defects (Se vacancies, antisite defects of Bi atoms in places of chalcogen atoms) with dominating Se vacancies (as the investigated material shows *n*-type conduction, and antisite defects should result in *p*-type conduction). The implementation of Cl in place of chalcogen in Bi_2_Te_3–*x*_Se_*x*_ probably leads to the two simultaneous effects. The first one is connected with the increase of the carrier concentration due to extra electrons introduced by Cl atoms. The second effect can be responsible for the restriction of the formation of both types of defects (chalcogen vacancies and antisite defects), as proposed by Kim et al.^56^ On the other hand, Cl oppositely can facilitate the formation of antisite defects due to the large electronegativity and small size of the Cl atoms leading to better charge balancing between vacancies and antisite defects. Nevertheless, both effects (the limited formation of defects or charge balancing) would result in enhanced mobility.

Assuming that one chlorine atom donates one electron to the carrier transport, we verify the effectiveness of this dopant in *n*-Bi_2_Te_3–*x*_Se_*x*_ alloys. This estimation helped us to see that ∼76–98% of the Cl atoms actively participate in electronic transport, suggesting Cl as the effective dopant for attuning the electric transport in *n*-Bi_2_Te_3–*x*_Se_*x*_ for high thermoelectric performance (Figure S4, Supporting information). The difference between the nominal carrier concentration of Cl and Hall concentration can be attributed to the effect of native defects in Bi_2_Te_3_-Bi_2_Se_3_ solid solutions, which is discussed above.

To verify the discussed above suggestions, we checked again the Hall data of the investigated Bi_2_Te_3–*x*–*y*_Se_*x*_Cl_*y*_ alloys considering three samples: I (*x* = 0.6, *y* = 0), II (*x* = 0.6, *y* = 0.015), and III (*x* = 0.6, *y* = 0.03). For these three specimens, the carrier concentrations are I: 5.8 × 10^19^ cm^–3^, II: 7.0 × 10^19^ cm^–3^, and III: 1.8 × 10^20^ cm^–3^, respectively. Therefore, the difference between the carrier concentration of sample I and sample II is Δ*n*_I–II_ = *n*_II_ – *n*_I_ = 1.2 × 10^19^ cm^–3^, and in the case of the samples with *y* = 0.015 and 0.03, the carrier concentration increases by Δ*n*_II–III_ = *n*_III_ – *n*_II_ = 11 × 10^19^ cm^–3^, which is a much higher value. As a result, assuming that one electron corresponds to one center, it is possible to roughly estimate the concentration of the charge scattering centers as Δ*n*_II–III_ – Δ*n*_I–II_*=* 9.8 × 10^19^ cm^–3^. Therefore, the effective change of the carrier concentration in our samples was observed when all charge scattering centers were compensated.

**Figure 5:**
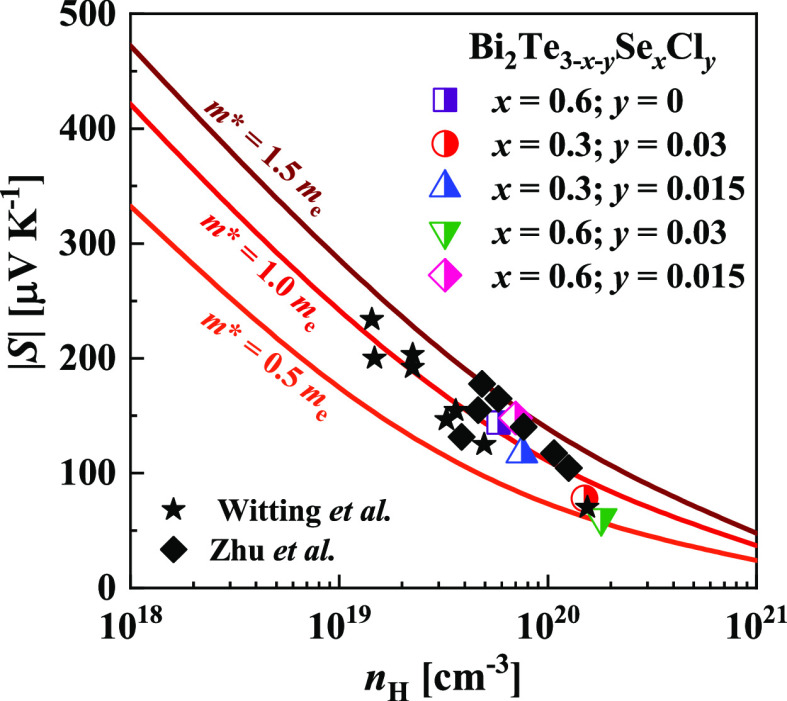
Seebeck coefficient as a function of carrier concentration at 298 K. Curves are calculated using the Kane band model. Filled (black) symbols indicate *n*-Bi_2_Te_3_ based materials taken from the literature for comparison.^57,58^

The Pisarenko plot of the Seebeck coefficient as a function of the carrier concentration is shown in Figure 5. All details of the performed calculations can be found in our previous papers.^59,60^ The obtained dependence of *S*(*n*) is in agreement with the previously published data.^57,58^ The density of electronic states (DOS) effective masses of the Bi_2_Te_3–*x*–*y*_Se_*x*_Cl_*y*_ alloys are also adjusted with the reported range of values (Table 2 and Figure 5).

**Figure 6:**
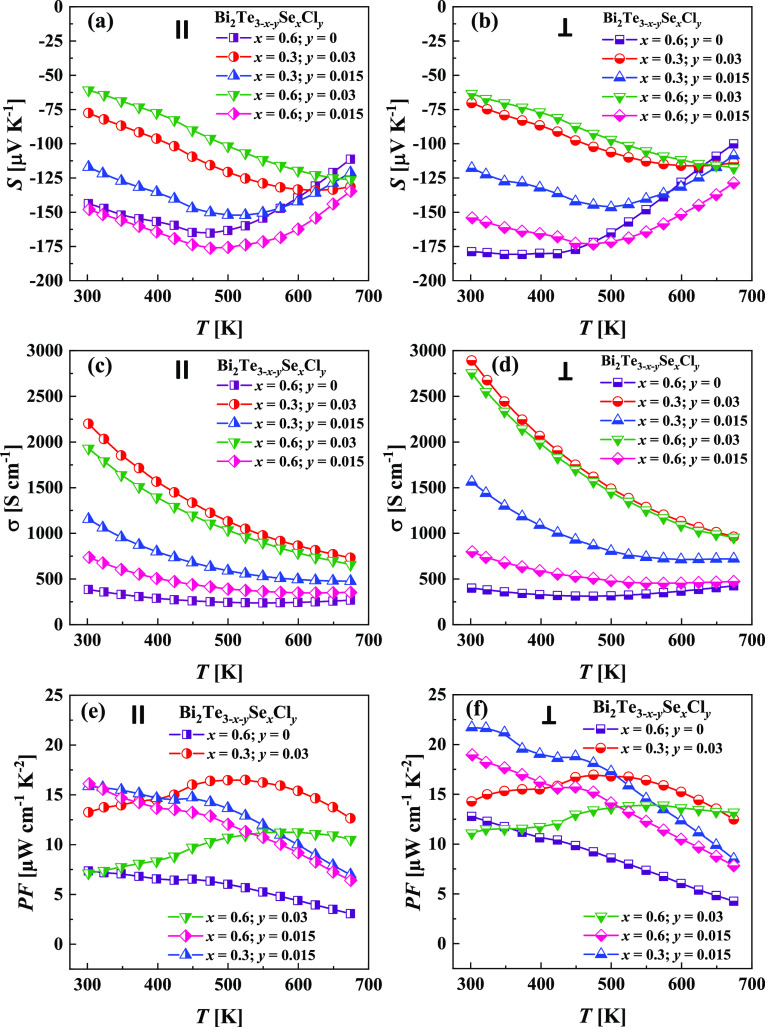
(a, b) Seebeck coefficient, (c, d) electrical conductivity, and (e, f) power factor as a function of temperature for Bi_2_Te_3–*x*–*y*_Se_*x*_Cl_*y*_ specimens measured (a, c, e) in parallel and (b, d, f) perpendicularly to the pressing direction.

The Seebeck coefficients measured with the help of commercial apparatus (Nemesis) at 298 K are comparable with the average *S* values estimated by the scanning thermoelectric microprobe for each particular sample (Table 2 and Figure S2a–e). The difference in the values can be connected with some features of the STMp measurements. Particularly, the needle during the STMp measurements is pressing the surface of the material with different pressures due to hardness fluctuation and the presence of the microscopic defects.^61^ The values of the Seebeck coefficient are negative over the entire temperature range, indicating electrons as the majority carriers for the investigated samples (Figure 6a,b). The temperature trends of the Seebeck coefficient *S* for Bi_2_Te_3–*x*–*y*_Se_*x*_Cl_*y*_ samples are very similar measured in parallel and perpendicular to the pressure direction. The Seebeck coefficient for the heavily doped Bi_2_Te_3–*x*–*y*_Se_*x*_Cl_*y*_ (*x* = 0.3, *y* = 0.03 and *x* = 0.6, *y* = 0.03) samples increases over the entire temperature range of 298–673 K. This observation can be connected with the high carrier concentration and localization of chemical potential deep in the conduction band. *S* values for the Cl-free and lightly Cl-doped Bi_2_Te_3–*x*–*y*_Se_*x*_Cl_*y*_ (*x* = 0.6, *y* = 0; *x* = 0.3, *y* = 0.015; and *x* = 0.6, *y* = 0.015) specimens show the bell-shaped form of *S*(*T*) due to the minority carrier effect at elevated temperatures, which is typical for narrow band gap semiconductors.^62^

Electrical conductivity as a function of temperature decreases over the investigated temperature range, indicating a metallic-like behavior, as depicted in Figure 6c,d. The Cl-free Bi_2_Te_3–*x*_Se_*x*_ sample shows the lowest trend of σ(*T*) due to the lowest carrier concentration *n* and carrier mobility μ. The low carrier concentration also results in the most significant effect of the intrinsic carrier transport at higher temperatures for this sample. The electrical conductivity measured in the direction perpendicular to the pressing direction is somewhat higher than the σ values measured for the same sample in the direction parallel to the pressing. This observation is in agreement with the previously reported data^63,64^ and suggests that the charge transport along crystal layers is better than across them.

Figure 6e,f represents the temperature trends of the power factor for the Bi_2_Te_3–*x*–*y*_Se_*x*_Cl_*y*_ samples. The best PF obtained in this work reaches the high value of 22 μW cm^–1^ K^–2^ in the direction perpendicular to pressing for the *n*-type Bi_2_Te_3–*x*_Se_*x*_Cl_*y*_ material with *x* = 0.3 and *y* = 0.015.

**Figure 7:**
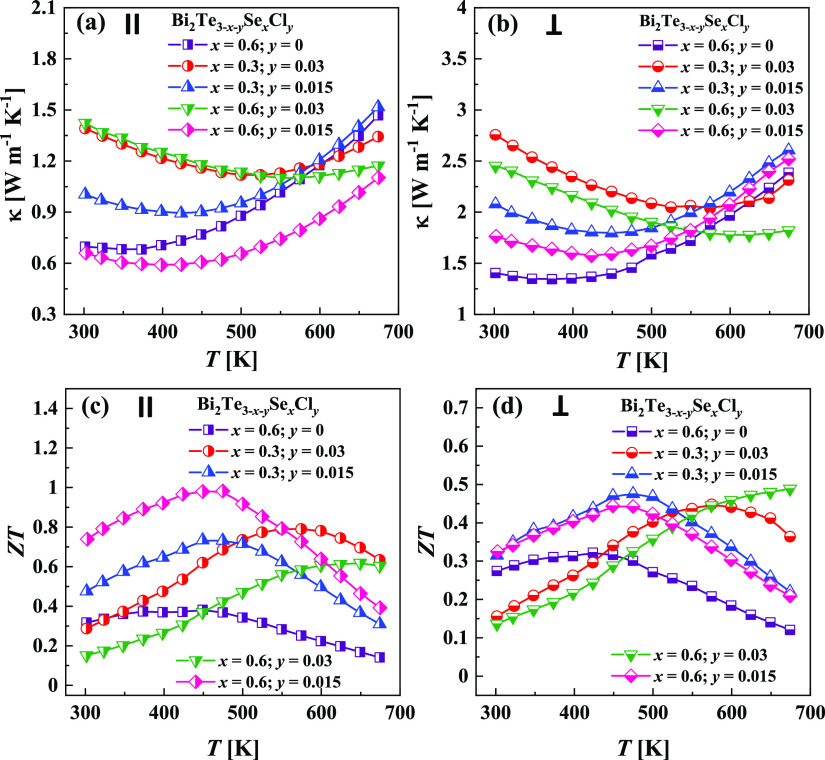
(a, b) Thermal conductivity κ and (c, d) dimensionless figure of merit ZT as a function of temperature for Bi_2_Te_3–*x*–*y*_Se_*x*_Cl_*y*_ specimens measured (a, c) in parallel and (b, d) perpendicularly to the pressing direction.

The total thermal conductivities of the Bi_2_Te_3–*x*–*y*_Se_*x*_Cl_*y*_ alloys measured in the directions perpendicular and parallel to the pressing axis show a significant difference for all the investigated samples, as is depicted in Figure 7a,b. A similar observation was also found in ref (35) for stochiometric Bi_2_Te_3_, where it was attributed to the crystal structure complexity,^18^ which will be discussed in the next section. The lowest thermal conductivity κ obtained in this work reaches the value of 0.6 W m^–1^ K^–1^. The observed sharp increase of the thermal conductivity at high temperatures in Cl-free and lightly Cl-doped samples can be connected with the intrinsic (bipolar) conduction regime, which can also be found in the temperature dependence of the electrical conductivity of those samples.

The dimensionless thermoelectric figure of merit ZT values as a function of temperature in the directions perpendicular and parallel to the pressing for the Bi_2_Te_3–*x*–*y*_Se_*x*_Cl_*y*_ samples are shown in Figure 7c,d. The ZT values for the samples measured in parallel were significantly higher than the ZT values measured perpendicularly to the pressing axis due to the differences in the thermal conductivities κ. The maximum thermoelectric figure of merit ZT obtained in this work reaches the value of ∼1.0, which is very high for the polycrystalline *n*-type Bi_2_Te_3–*x*_Se_*x*_ alloys. The maximum thermoelectric figure of merit ZT obtained in this work reaches the value of ∼1.0, which is reasonably high for the polycrystalline *n*-type Bi_2_Te_3–*x*_Se_*x*_ alloys. This value is in the range of ZT = 0.85–1.2, which are the best ZTs reported in *n*-type Bi_2_Te_3–*x*_Se_*x*_ alloys.^54,55^ The further optimization of the power factor of Bi_2_Te_3–*x*_Se_*x*_ by Cl doping can lead to an even higher thermoelectric figure of merit, opening practical interest for the developed materials. Moreover, the maximum ZT corresponds to different temperatures (e.g., 473 K for *x* = 0.6, *y* = 0.015; and 573 K for *x* = 0.3, *y* = 0.03 (Figure 7c)), thus making this system a very promising candidate for the construction of a functionally graded TE leg.

### 3.3 Thermal Conductivity

Thermal conductivity of the narrow band gap TE materials is represented as a sum of the electronic κ_e_, lattice κ_L_, and bipolar κ_B_ components of the thermal conductivity (κ = κ_L_ + κ_e_ + κ_B_). Electronic heat transport usually follows the Wiedemann–Franz law (κ_e_ = *L*σ*T*, where *L* is the Lorenz number) and cannot be simply engineered as κ_e_ is linearly proportional to the electrical conductivity σ*.* Therefore, the suppression of the bipolar conduction and decrease of the lattice thermal conductivity remain the main methods of lowering the total thermal conductivity in TE materials. In the case of the Bi_2_Te_3_-based alloys, the bipolar conduction deteriorates sharply the thermoelectric performance even at moderately high temperatures due to the narrow band gap, the high carrier mobility, and unique features of the band structure.^21,65^

The first step in understanding the origins of heat transport in TE materials is the accurate determination of the κ_e_, κ_L_, and κ_B_. As most TE materials pose heavily degenerated semiconducting properties, the main problem here is connected with the accurate calculations of the Lorenz number *L* and accounting of the bipolar conduction. With the aim to evaluate the electronic, lattice, and bipolar components from the total thermal conductivity, we employed the two-band Kane model for the investigated Bi_2_Te_3–*x*–*y*_Se_*x*_Cl_*y*_ alloys. The utilization of the two-band Kane model (particularly the consideration of conduction and valence bands) seems to be justified by the necessity to take into account the contribution of holes in the valence band, which in turn allows for the calculation of the bipolar thermal conductivity and a decrease of the absolute value of the Seebeck coefficient at high temperatures observed during measurements.

**Figure 8:**
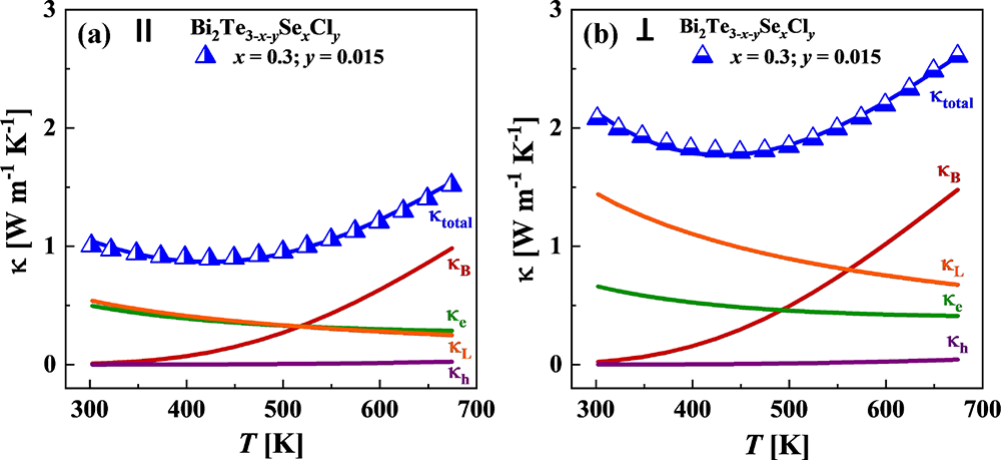
The relative magnitude of the lattice κ_L_, hole κ_h_, electronic κ_e,_ and bipolar κ_B_ components in the total thermal conductivity κ estimated (a) in parallel and (b) perpendicularly to the pressing direction. Curves are obtained using the two-band Kane model results of Bi_2_Te_3_ trends with temperature and based on fits of the Bi_2_Te_3–*x*–*y*_Se_*x*_Cl_*y*_ (*x* = 0.3, *y* = 0.015) sample.

The utilized model is based on the methodology described by Witting et al.^57^ In short, the approach is based on the simultaneous fitting of the two-band Kane model to the experimentally obtained values of *S*, σ, and κ. The free parameters of the model include the band gap energy, the acoustic deformation potential, carrier effective masses and mobilities of electrons in the conduction band and holes in the valence band, the lattice thermal conductivity at 298 K, and the lattice thermal conductivity exponent. The main difference between the approaches employed by Witting et al. and those utilized in this work is the use of the Kane band model, given by eqs S1–S12,^66−68^ which results in a better agreement between the fitted and experimental values of thermoelectric parameters. After the fitting, it is possible to separate the contributions of majority and minority carriers on the Seebeck coefficient and electrical conductivity as shown in Figure S5, as well as the electron, hole, bipolar, and lattice contributions to thermal conductivity as shown in Figure 8.

**Figure 9:**
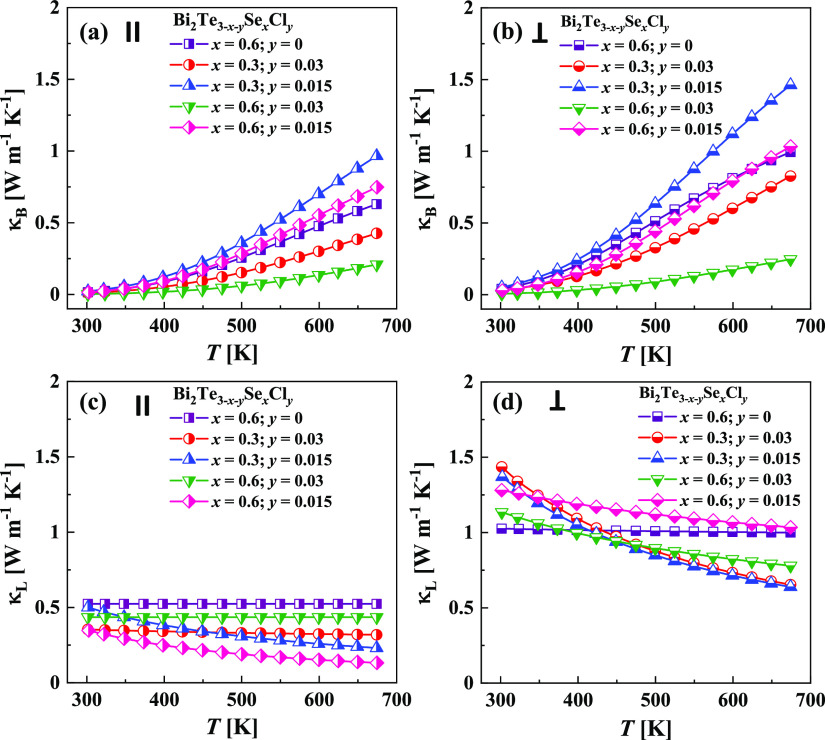
(a, b) Bipolar thermal conductivity κ_B_ and (c, d) lattice thermal conductivity κ_L_ as a function of temperature for Bi_2_Te_3–*x*–*y*_Se_*x*_Cl_*y*_ specimens, measured (a, c) in parallel and (b, d) perpendicularly to the pressing direction. Calculations were performed using the two-band Kane model.

Results of the analysis of the thermal conductivity within the two-band Kane model in different pressing directions are shown in Figure 8a,b, taking the Bi_2_Te_2.685_Se_0.3_Cl_0.015_ sample as an example. The temperature-dependent bipolar κ_B_ and lattice κ_L_ thermal conductivity values measured in different pressing directions for all investigated alloys are shown in Figure 9. The obtained values of lattice thermal conductivity for samples measured in parallel to the pressing direction (∼0.3–0.5 W m^–1^ K^–1^ at 298 K) (Figure 9c) were around 3 times lower than κ_L_ for samples measured perpendicularly to the pressing direction (∼1.0–1.5 W m^–1^ K^–1^ at 298 K) (Figure 9d). To understand such a drastic difference in lattice thermal conductivity between the two directions, we combined the results of the transport coefficient calculations with ultrasonic measurements and analysis of chemical bonding in Bi_2_Te_3–*x*_Se_*x*_ alloys.

As we can see in Figures 8 and 9, the κ_B_ values at room temperature are very small for all investigated samples, indicating a weak carrier excitation at this temperature. With the rising temperature, κ_B_ sharply increases due to thermally induced excitation of the electron–hole pairs across the band gap and extra heat being released as a result of electron–hole recombination. Samples measured perpendicularly to the pressing direction generally have a higher bipolar component of κ due to the effect of texturization and easier excitation of electron–hole pairs across the band gap in this direction of the Brillouin zone.

The selected combination of materials also gives us the possibility to verify the individual effect of the Se alloying and Cl doping on the bipolar thermal conductivity. Bi_2_Te_3–*x*–*y*_Se_*x*_Cl_*y*_ samples with *x* = 0.6 show smaller values of κ_B_ in both pressing directions than samples with *x* = 0.3 (if the content of Cl is the same). This observation can be connected with a wider band gap (*E*_g_ = 0.25 eV) in materials with *x* = 0.6 compared to the materials with *x* = 0.3 (*E*_g_ = 0.2 eV),^33,57^ which is known to inhibit the creation of electron–hole pairs that contribute to bipolar thermal conductivity. On the other hand, in the samples with the same content of Se but different amounts of Cl, it can be suggested that Cl strongly suppresses the bipolar conduction through an increase in the ratio of majority to minority carriers. To evaluate this effect, let us again analyze Bi_2_Te_3–*x*–*y*_Se_*x*_Cl_*y*_ alloys with I (*x* = 0.6, *y* = 0), II (*x* = 0.6, *y* = 0.015), and III (*x* = 0.6, *y* = 0.03). On the one hand, the change in temperature trends of the bipolar conduction for material I and material II is small due to the carrier compensation effect, which is discussed by analyzing the measured Hall data for these samples. On the other hand, the κ_B_ values for sample III are much smaller than in the case of sample II, indicating the effective suppression of the intrinsic transport by the halogen dopant through the tuning of the chemical potential.^69^ Moreover, the same conclusion about the strong suppressing of the bipolar conduction is also evident from the analysis of the Bi_2_Te_3–*x*–*y*_Se_*x*_Cl_*y*_ samples with *x* = 0.3 and different contents of chlorine (*y* = 0.015 and 0.03).

The lattice thermal conductivity was obtained by subtracting the electronic κ_e_ and bipolar κ_B_ components from the total thermal conductivity (Figure 9c,d). The temperature trends of the lattice thermal conductivity for all Bi_2_Te_3–*x*–*y*_Se_*x*_Cl_*y*_ samples are decreasing over the investigated temperature range. The slope of this decrease is different for the samples with different contents of selenium (e.g., *x* = 0.3 and 0.6), which can be an indicator of the different dominance of the phonon scattering mechanisms. The lattice thermal conductivities of the samples measured in parallel to the pressing direction show significantly lower values compared to the samples measured perpendicularly to the pressing direction. In this work, the lowest lattice thermal conductivity was obtained for the Bi_2_Te_2.385_Se_0.6_Cl_0.015_ sample in the direction parallel to pressing. For this sample, κ_L_ decreased from 0.4 W m^–1^ K^–1^ at 298 K to an ultralow value of 0.15 W m^–1^ K^–1^ at 673 K, which is even below the minimum thermal conductivity, as shown in Tables 3 and 4. The calculations based on the two-band Kane model have been performed by taking into account as much experimental data as possible to robustly grasp the complexity of the interactions between the thermoelectric properties in the studied materials. However, it should be noted that some assumptions, such as the decrease of the lattice thermal conductivity given a priori by eq. S11 or constant electron and hole effective masses in the discussed temperature range, had to be made to facilitate the calculations. Those assumptions could have an effect on the obtained values of the thermoelectric properties and lead to the underestimation of the lattice thermal conductivity.

**Table 3:** Elastic and Thermal Transport Properties for Bi_2_Te_3–*x*–*y*_Se_*x*_Cl_*y*_ Specimens Measured in Parallel to the Pressing Direction

Bi_2_Te_3–*x*–*y*_Se_*x*_Cl_*y*_	*v*_l_, ms^–1^	*v*_t_, ms^–1^	*v*_m_, ms^–1^	Θ_D_, K	ν	γ	*l*_ph_, Å	κ_glass_, Wm^–1^ K^–1^	κ_diff_, Wm^–1^ K^–1^
*x* = 0.6, *y* = 0	2667	1519	1688	158.5	0.26	1.55	10.03	0.31	0.19
*x* = 0.3, *y* = 0.03	2570	1522	1686	152.9	0.23	1.41	6.90	0.30	0.19
*x* = 0.3, *y* = 0.015	2592	1503	1668	150.8	0.25	1.49	9.87	0.30	0.19
*x* = 0.6, *y* = 0.03	2536	1543	1705	156.2	0.21	1.31	8.23	0.31	0.19
*x* = 0.6, *y* = 0.015	2568	1516	1680	152.2	0.23	1.42	6.67	0.30	0.19

**Table 4:** Elastic and Thermal Transport Properties for Bi_2_Te_3–*x*–*y*_Se_*x*_Cl_*y*_ Specimens Measured Perpendicularly to the Pressing Direction

Bi_2_Te_3–x–y_Se_x_Cl_y_	*v*_l_, ms^–1^	*v*_t_, ms^–1^	v_m_, ms^–1^	Θ_D_, K	ν	γ	*l*_ph_, Å	κ_glass_, Wm^–1^ K^–1^	κ_diff_, Wm^–1^ K^–1^
*x* = 0.6, *y* = 0	2855	1496	1673	155.3	0.31	1.84	19.83	0.32	0.20
*x* = 0.3, *y* = 0.03	2806	1496	1672	154.9	0.30	1.78	28.20	0.31	0.20
*x* = 0.3, *y* = 0.015	2786	1524	1700	157.3	0.29	1.69	26.50	0.31	0.20
*x* = 0.6, *y* = 0.03	2819	1513	1690	157.4	0.30	1.76	21.64	0.32	0.20
*x* = 0.6, *y* = 0.015	2817	1539	1716	159.6	0.29	1.70	24.10	0.32	0.20

To gain insight into the lattice thermal conductivity of the Bi_2_Te_3–*x*–*y*_Se_*x*_Cl_*y*_ alloys, we performed ultrasonic measurements. Tables 3 and 4 show the obtained values of the longitudinal *v*_l_, transverse *v*_t_, and the average *v*_m_ speed of sound; Debye temperatures Θ_D_; the Poisson ratio ν; Grüneisen parameter γ; phonon mean free path *l*_ph_; and the minimum thermal conductivity κ_glass_ and κ_diff_ for Bi_2_Te_3–*x*–*y*_Se_*x*_Cl_*y*_ alloys.

The measured speed of sound values are in the range of the previously reported data for the Bi_2_Te_3_-based alloys.^70^ The obtained low values of the Debye temperature (∼155–160 K) suggest extremely weak chemical bonding and slow phonon propagation in the investigated alloys. A large difference between the longitudinal speed of sound, which was measured in parallel and perpendicularly to the pressing direction, is in line with the anisotropic properties of Bi_2_Te_3_-based alloys. The performed investigations show that the propagation of phonons along the quintuple layers is faster than across the van der Waals gaps. The values of the mean free paths of phonons are also shorter in parallel to the pressing direction than perpendicular to it.

On the one hand, the low values of κ_L_ obtained in this work can be explained by the conventional concept of the fabrication of solid solutions Bi_2_Te_3_/Bi_2_Se_3._^71^ The electrons in solid solutions are scattered on the defects, which lead to the diminishing role of the high-frequency phonons in the transport of heat. Most of the heat flux, in this case, is transported through the low-frequency phonons, and as they can interact with the electrons, the role of phonon–electron scattering in heat transport in the solid solutions becomes significantly larger. The other possible reason for the suppressed phonon transport can be connected with the large mass fluctuation between Cl, Te, and Se atoms. Some decrease in the κ_L_ is also expected due to the (Te/Se) inhomogeneities observed in the SEM and STMs images. Nevertheless, all the above explanations can tell us nothing about the origins of the very different κ_L_ values in different directions, thus forcing us to look for the explanation of this phenomenon in the anisotropy of properties of the Bi_2_Te_3_-based alloys.

**Figure 10:**
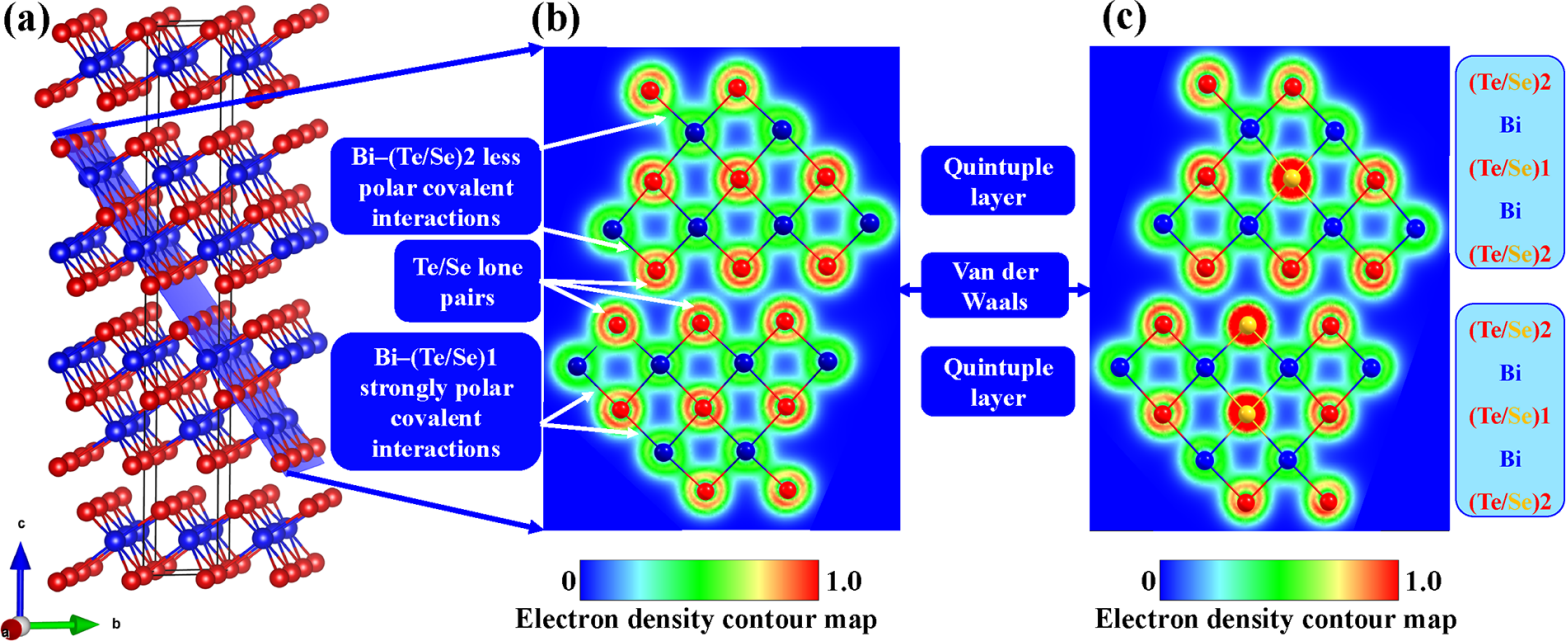
(a) Crystal structure unit of the Bi_2_Te_3–*x*_Se_*x*_ with the indicated atomic plane of the electron density contour map of (b) Bi_2_Te_3_ and (c) Bi_2_Te_3–*x*_Se_*x*_.

To further understand the chemical bonding environment in Bi_2_Te_3–*x*–*y*_Se_*x*_Cl_*y*_, we calculated the electron density contour map (EDC), which is a measure of the electron localization in atomic or molecular systems.^20^ In agreement with the interatomic distances, the electron density contour maps show that Bi-(Te/Se)1 bonds are more polar than Bi-(Te/Se)2 bonds (Figure 10), as earlier reported for undoped Bi_2_Te_3_ by Grin.^18^ Such bonding inhomogeneity is even more prominent in Bi_2_Te_3–*x*_Se_*x*_ alloys due to the presence of different chalcogen atoms of Te and Se (Figure 10c). These [(Te/Se)-Bi-(Te/Se)-Bi-(Te/Se)] quintuple layers with polar covalent interactions are divided by the van der Waals gaps with weak interactions of (Te/Se) lone pair electrons. Therefore, the low lattice thermal conductivity in both pressing directions is connected with the different polarities of Bi-(Te/Se)1 and Bi-(Te/Se)2, while the lone-pair (Te/Se) interactions are mainly responsible for the extremely low lattice thermal conductivity in parallel to the pressing direction.

## 4 Conclusions

In summary, we have successfully optimized the thermoelectric properties of the *n*-type Bi_2_Te_3–*x*_Se_*x*_ alloys at elevated temperatures by Cl doping. Like the other halogen dopants in Bi_2_Te_3_-based alloys, chlorine tunes the carrier concentration and effectively suppresses the intrinsic excitation, which leads to a reduced bipolar conductivity, as it was evaluated using the developed two-band Kane model. The estimated ultralow lattice thermal conductivity (as low as 0.15 Wm^–1^ K^–1^ at 673 K) of Cl-doped Bi_2_Te_3–*x*_Se_*x*_ measured in parallel to the pressing direction is explained by the Bi-(Te/Se)1 and Bi-(Te/Se)2 bonding inhomogeneity and (Te/Se) lone-pair interactions. The combination of the suppressed bipolar, low lattice thermal conductivity, and optimized electronic transport properties results in a maximum ZT of 1.0 at 573 K for the *n*-type Bi_2_Te_3–*x*–*y*_Se_*x*_Cl_*y*_ alloy with *x* = 0.6 and *y* = 0.015. Moreover, the high ZT parameter was obtained at different temperatures, thus opening the potential to use the developed *n*-type Bi_2_Te_3_-based materials for the fabrication of the functionally graded thermoelectric leg.

## Supporting Information Available

Powder XRD patterns of samples after synthesis; scanning thermoelectric microprobe data; details of the two-band Kane model calculations of the thermoelectric properties; and details of the elastic and thermal transport property calculations (PDF)
