# Supplementary material for: Ultralow Lattice Thermal Conductivity and Improved Thermoelectric Performance in Cl-Doped Bi2Te3–xSex Alloys
Source: ACS Appl Mater Interfaces. 2022 Jul 13;14(29):33567–79. doi: 10.1021/acsami.2c08686 (PMC9335406; doi:10.1021/acsami.2c08686)
Supplement: Supplementary file 1 — am2c08686_si_001.pdf [file am2c08686_si_001.pdf]

Supporting Information

Ultralow lattice thermal conductivity and improved thermoelectric performance in Cl-doped $Bi_2Te_{3-x}Se_x$ alloys

Taras Parashchuk[1,&], Rafal Knura[1,2,&], Oleksandr Cherniushok[1], and Krzysztof T. Wojciechowski[1,*]

[1]Thermoelectric Research Laboratory, Department of Inorganic Chemistry, Faculty of Materials Science and Ceramics, AGH University of Science and Technology, Mickiewicza Ave. 30, 30-059 Krakow, Poland
[2]Department of Science, Graduate School of Science and Technology, Kumamoto University, 2 Chome-39-1 Kurokami, Chuo Ward, 860-8555 Kumamoto, Japan

[*]Corresponding author: wojciech@agh.edu.pl

Table of contents

Figure S1. X-ray diffraction patterns of the mixed with LaB_6 standard $Bi_2Te_{3\text{-}x\text{-}y}Se_xCl_y$ powdered ingots, which were used for cell parameters determination.

Figure S2. Distribution and histogram of Seebeck coefficient for $Bi_2Te_{3\text{-x-y}}Se_xCl_y$ specimens after LFA: (a) $x = 0.6$, $y = 0.015$; (b) $x = 0.6$, $y = 0.03$; (c) $x = 0.3$, $y = 0.015$; (d) $x = 0.3$, $y = 0.03$; (e) $x = 0.6$, $y = 0$.

Figure S3. The lattice thermal conductivity and bandgap as a function of x in $Bi_2Te_{3\text{-}x}Se_x$ solid solution.

Figure S4. Correlation between the Cl dopant concentration and carrier concentration obtained from the Hall effect measurements. The value of the slope close to 1 indicates that every chlorine atom contributes one electron to the conduction band.

Figure S5. The relative magnitude of the Seebeck coefficient caused by electron and hole components in the total values of S estimated parallelly (a) and perpendicularly (b) to the pressing direction. The relative magnitude of the electrical conductivity caused by electron and hole components in the total values of σ estimated parallelly (a) and perpendicularly (b) to the pressing direction. Curves are obtained using the two-band Kane model results of Bi_2Te_3 trends with temperature and based on fits of the $Bi_2Te_{3\text{-}x\text{-}y}Se_xCl_y$ ($x = 0.3$, $y = 0.015$) sample.

Equations S1-S12. Details of the two-band Kane model calculations of the thermoelectric properties;

Equations S13-S19. Details of elastic properties calculations.

Equations S20-S21. Details of thermal transport properties calculations.

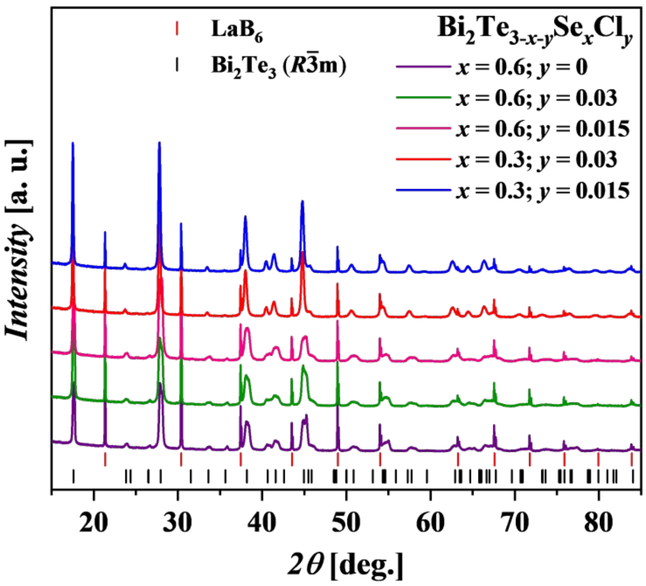

Figure S1. X-ray diffraction patterns of the mixed with LaB_6 standard $Bi_2Te_{3-x-y}Se_xCl_y$ powdered ingots, which were used for cell parameters determination.

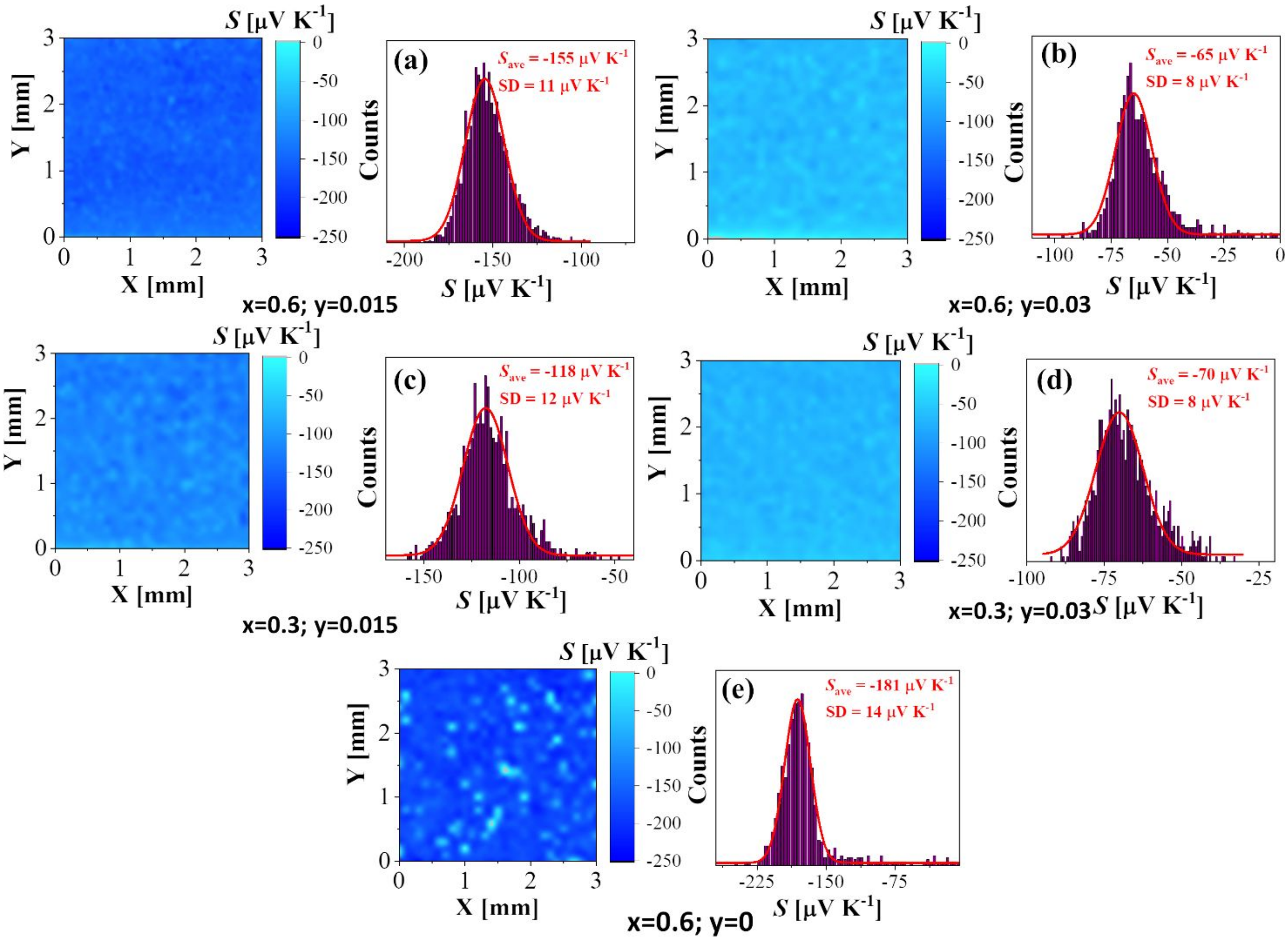

Figure S2. Distribution and histogram of Seebeck coefficient for $Bi_2Te_{3-x-y}Se_xCl_y$ specimens after LFA: (a) $x = 0.6$, $y = 0.015$; (b) $x = 0.6$, $y = 0.03$; (c) $x = 0.3$, $y = 0.015$; (d) $x = 0.3$, $y = 0.03$; (e) $x = 0.6$, $y = 0$.

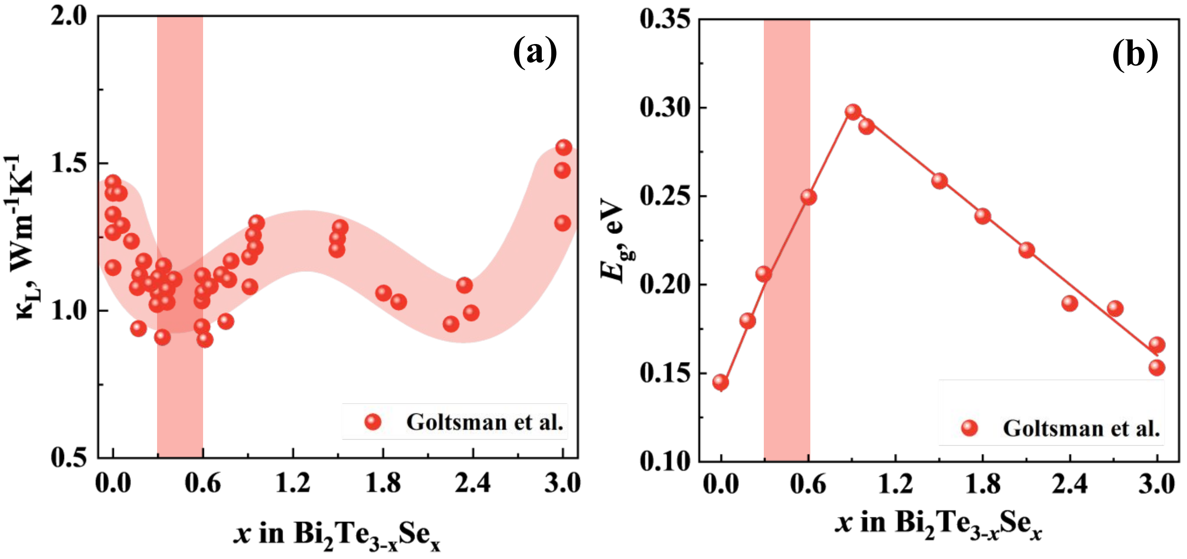

Figure S3. The lattice thermal conductivity and bandgap as a function of x in $Bi_2Te_{3-x}Se_x$ solid solution. The graphs were constructed using data available in [1].

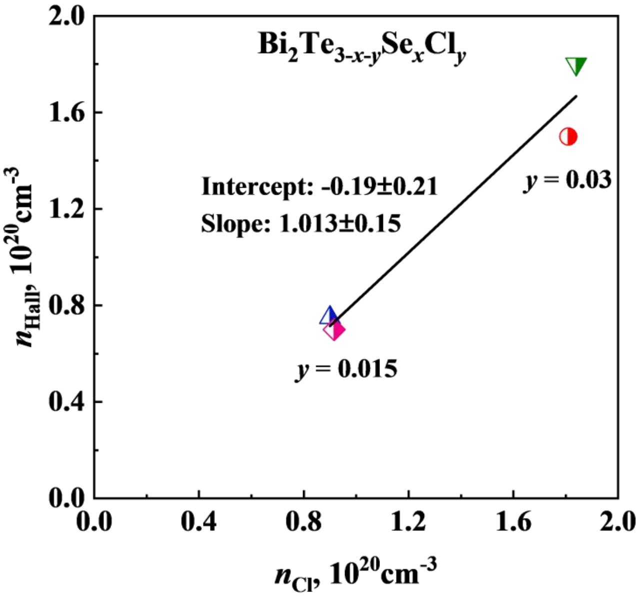

Figure S4. Correlation between the Cl dopant concentration and carrier concentration obtained from the Hall effect measurements. The value of the slope close to 1 indicates that every chlorine atom contributes one electron to the conduction band.

Two-band Kane model

The Kane band model for single carrier type, which is a foundation of the utilized approach, is based on the following equations used for calculation of the material properties such as the carrier concentration – n, the Seebeck coefficient – S, the electrical conductivity – σ, the Lorenz number – L and the carrier contribution to thermal conductivity – κ_{carrier} [2-4].

$$n = \frac{\left(2m^* k_B T\right)^{3/2}}{3\pi^3 \hbar^3}\left({}^0F_0^{3/2}(\eta)\right), \tag{S1}$$

$$S = -\frac{k_B}{e}\left(\frac{{}^1F_{-2}^1(\eta)}{{}^0F_{-2}^1(\eta)} - \eta\right), \tag{S2}$$

$$\sigma = \frac{e^2}{m^*}\frac{2\hbar\rho v^2}{3\pi E_{ac}^2}\left({}^0F_{-2}^1(\eta)\right), \tag{S3}$$

$$L = \left(\frac{k_B}{e}\right)^2\left[\frac{{}^2F_{-2}^1(\eta)}{{}^0F_{-2}^1(\eta)} - \left(\frac{{}^1F_{-2}^1(\eta)}{{}^0F_{-2}^1(\eta)}\right)^2\right], \tag{S4}$$

$$\kappa_{carrier} = L\sigma T, \tag{S5}$$

where k_B, $\hbar$, e, m^*, ρ, v, E_{ac}, T denote the Boltzmann constant, the Planck's constant, electron charge, effective mass at band extremum, material's density, material's speed of sound, acoustic phonon deformation potential and temperature, respectively; F is an integral given by:

$$ {}^nF_k^m(\eta) = \int_0^\infty \left(-\frac{\partial f(\eta)}{\partial \varepsilon}\right)\varepsilon^n\left(\varepsilon + \alpha\varepsilon^2\right)^m\left(1 + 2\alpha\varepsilon\right)^k d\varepsilon, \tag{S6}$$

where $f(\eta)$ is the Fermi-Dirac integral, which is a function of the reduced Fermi level (η) and reduced energy $\left(\varepsilon = \frac{E}{k_B T}\right)$, and α is a nonparabolicity parameter $\left(\alpha = \frac{k_B T}{E_g}\right)$.

Due to the narrow bandgap $E_g < 0.3$ eV, in order to fully explain the temperature dependence of thermoelectric properties such as the decrease of the absolute value of the Seebeck coefficient or

the bipolar thermal conductivity at high temperatures, it was necessary to take into account the influence of minority carriers. This was achieved by introducing the contribution of holes in the valence band through the following equations [3-4]:

$$n = \sum_i n_i \,, \tag{S7}$$

$$\sigma = \sum_i \sigma_i \,, \tag{S8}$$

$$S = \frac{\sum_i \sigma_i S_i}{\sum_i \sigma_i} \,, \tag{S9}$$

$$\kappa_B = T\left(\sum_i \sigma_i S_i^2 + \frac{\left(\sum_i \sigma_i S_i \right)^2}{\sum_i \sigma_i} \right), \tag{S10}$$

$$\kappa_L = \kappa_{L,300K}\left(\frac{T}{300K} \right)^{-a}, \tag{S11}$$

$$\kappa = \sum_i \kappa_{carrier\,i} + \kappa_B + \kappa_L \,, \tag{S12}$$

where index *i* distinguishes majority and minority carriers; *n*, σ, *S* and *T* denote carrier concentration, electrical conductivity, the Seebeck coefficient, and temperature, respectively; κ_B, κ_L, $\kappa_{carrier,}$ and κ denote bipolar, lattice, carrier (electron or hole) contributions to thermal conductivity and total thermal conductivity, respectively. Finally, *a* is the exponential factor that governs the lattice thermal conductivity. Also, care must be taken to assign the correct sign convention to the Seebeck coefficient and charge carrier concentrations (electrons or holes).

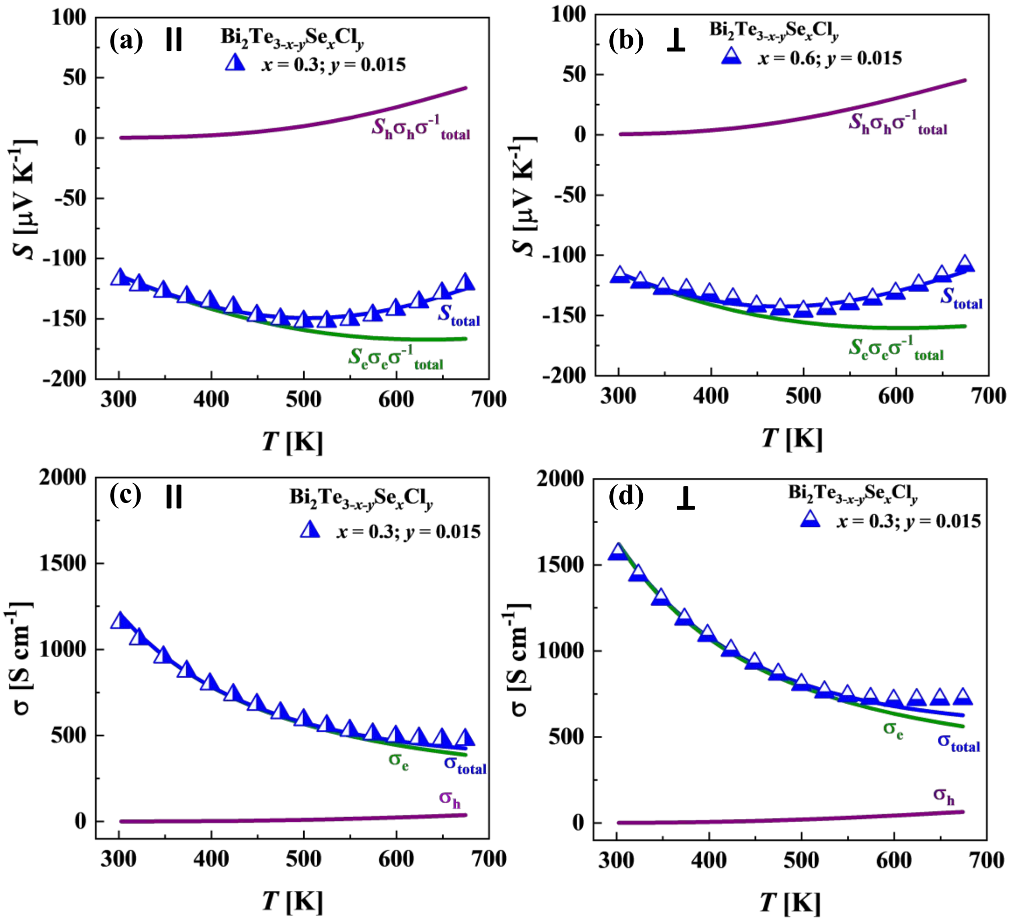

Figure S5. The relative magnitude of the Seebeck coefficient caused by electron and hole components in the total values of S estimated parallelly (a) and perpendicularly (b) to the pressing direction. The relative magnitude of the electrical conductivity caused by electron and hole components in the total values of σ estimated parallelly (a) and perpendicularly (b) to the pressing direction. Curves are obtained using the two-band Kane model results of Bi_2Te_3 trends with temperature and based on fits of the $Bi_2Te_{3\text{-}x\text{-}y}Se_xCl_y$ ($x = 0.3$, $y = 0.015$) sample.

Elastic properties

The bulk modulus was calculated using the following equation [4-6]:

$$B = \rho\left(\upsilon_L^2 - \frac{4}{3}\upsilon_T^2\right), \quad \text{(S13)}$$

where ρ is the material density.

The shear modulus was calculated as:

$$G = \upsilon_T^2 \rho. \tag{S14}$$

The Young's modulus was calculated as:

$$E = \frac{9BG}{3B + G}. \tag{S15}$$

The Poisson's ratio was calculated as:

$$\nu = \frac{E - 2G}{2G}. \tag{S16}$$

The Debye temperatures were calculated using the following expression [7]:

$$\Theta_D = \frac{h}{k_B}\left[\frac{3n}{4\pi}\left(\frac{N_A \rho}{M}\right)\right]^{1/3} v_m, \tag{S17}$$

where h is Planck's constant, k_B is Boltzmann's constant, N_A is Avogadro's number, M is the molecular weight, n is the number of atoms in the molecule, and v_m is the averaged wave velocity integrated over several crystal directions [7]:

$$v_m = \left[\frac{1}{3}\left(\frac{2}{v_t^3} + \frac{1}{v_l^3}\right)\right]^{-1/3}, \tag{S18}$$

where v_l and v_t are the longitudinal and transverse sound velocities.

Grüneisen parameters γ were calculated using the following equation [8]:

$$\gamma = \frac{3}{2}\left(\frac{1+\nu}{2-3\nu}\right). \tag{S19}$$

where ν is the Poisson ratio.

Thermal transport properties

From the kinetic theory, the lattice thermal conductivity is expressed as [9]:

$$\kappa_L = \frac{1}{3} C_V v_m l_{ph} \tag{S20}$$

where κ_L is experimental lattice thermal conductivity, C_V is the specific heat at constant volume, v_m is the average sound velocity. Considering this, the phonon mean free path can be calculated using the following equation [10]:

$$l_{ph} = \frac{3\kappa_L}{C_V v_m} \tag{S21}$$

For the calculation of l_{ph} at 298 K, the κ_L was estimated using the two-band Kane model within the procedure described above, specific heat capacity was estimated using the Dulong-Petit approximation, and average sound velocity was obtained from the acoustic data of longitudinal v_l and transverse v_t sound velocities using Equation S18.

References

[1] Goltsman, B. M.; Kudinov, V. A.; Smirnov, I. A. Thermoelectric Semiconductor Materials Based on Bi_2Te_3; Nauka: Moskow, 1972.

[2] Witting, I. T.; Chasapis, T. C.; Ricci, F.; Peters, M.; Heinz, N. A.; Hautier, G.; Snyder, G. J. The Thermoelectric Properties of Bismuth Telluride. Adv. Electron. Mater. 2019, 5 (6), 1800904. https://doi.org/10.1002/AELM.201800904.

[3] Naithani, H.; Dasgupta, T. Critical Analysis of Single Band Modeling of Thermoelectric Materials. ACS Appl. Energy Mater. 2020, 3 (3), 2200–2213. https://doi.org/10.1021/ACSAEM.9B02015/SUPPL_FILE/AE9B02015_SI_001.PDF.

[4] Y.I. Ravich, B.A. Efimova, I.A. Smirnov, Semiconducting Lead Chalcogenides, Springer US, 1970. https://doi.org/10.1007/978-1-4684-8607-0.

[5] B.M. Askerov, Electron Transport Phenomena in Semiconductors, WORLD SCIENTIFIC, 1994. https://doi.org/10.1142/1926.

[6] Thermoelectrics Handbook: Macro to Nano - 1st Edition - D.M. Rowe - H, (n.d.). https://www.routledge.com/Thermoelectrics-Handbook-Macro-to-Nano/Rowe/p/book/9780849322648 (accessed May 9, 2022).

[7] O.L. Anderson, A simplified method for calculating the debye temperature from elastic constants, J. Phys. Chem. Solids. 24 (1963) 909–917. https://doi.org/10.1016/0022-3697(63)90067-2.

[8] D.S. Sanditov, V.N. Belomestnykh, Relation between the parameters of the elasticity theory and averaged bulk modulus of solids, Tech. Phys. 56 (2011) 1619–1623. https://doi.org/10.1134/S106378421111020X.

[9] T. M. Tritt, Thermal conductivity: theory, properties, and applications, Springer Science & Business Media, 2005.

[10] H. Xie, S. Hao, S. Cai, T.P. Bailey, C. Uher, C. Wolverton, V.P. Dravid, M.G. Kanatzidis, Ultralow thermal conductivity in diamondoid lattices: high thermoelectric performance in chalcopyrite $Cu_{0.8+y}Ag_{0.2}In_{1-y}Te_2$, Energy Environ. Sci. 13 (2020) 3693–3705. https://doi.org/10.1039/D0EE02323J.
